# FRAMEWORKS OF THE RELATIONSHIP BETWEEN HIV AND AGING AMONG HIV CLINIC PROVIDERS: A QUALITATIVE STUDY

**DOI:** 10.1093/geroni/igac059.2831

**Published:** 2022-12-20

**Authors:** Madeline Villalba, Rachel Schenkel, Michael Yin, Peter Gordon, Abigail Baim-Lance

**Affiliations:** Icahn School of Medicine at Mount Sinai, New York, New York, United States; Emory University School of Medicine, Atlanta, Georgia, United States; Columbia University Medical Center, New York, New York, United States; Columbia University Medical Center, New York, New York, United States; Icahn School of Medicine at Mount Sinai, New York, New York, United States

## Abstract

As a result of advancements in antiretroviral therapy (ART), people living with HIV (PLWH) are living longer: it is estimated that 70% of PLWH in the US will be over the age of 50 by 2030, raising questions of what it means to age with HIV and how to care for these individuals most effectively. To qualitatively explore how providers who care for older PLWH and other comorbidities conceptualize the relationship between HIV and aging, we conducted 11 semi-structured interviews with physicians, RN case managers, and administrators at two health systems’ HIV clinics in New York City and the Hudson Valley between November 2019 and July 2020. We coded the interviews, developing a codebook through an iterative process, and thematically analyzed the data. Analysis revealed a range of interrelationships between HIV, aging, and comorbidities across pathophysiological and psychosocial dimensions. Providers commonly espoused that, for older PLWH, comorbidities are comparatively more difficult to manage than HIV and constitute the primary driver of pathophysiology and/or mortality. In contrast, providers viewed patients as regarding HIV as more deadly and engaging with HIV care more diligently, which providers related to patients’ long-term experiences of living through the HIV/AIDS epidemic. Providers’ comorbidity-centric framings of the clinical relationship between HIV, aging, and comorbidities mark a departure from HIV-dominant forms of thinking. Our findings have important implications for interdisciplinary care management and suggest how to support the development of asset-based approaches to encourage patient engagement with comorbidity care based on fidelity to HIV regimens.

